# CD137: A Member of the TNFR Family - in Psoriasis Skin Lesions in Comparison with Normal Skin Specimens

**DOI:** 10.30699/IJP.2020.118767.2290

**Published:** 2020-12-20

**Authors:** Parvin Rajabi Dehnavi, Seyyed Mehdi Eftekhari, Azadeh Kadkhodaei, Amirhosein Kefayat

**Affiliations:** 1 *Department of Pathology, Faculty of Medical Sciences, Isfahan University of Medical Sciences, Isfahan, Iran*; 2 *Cancer Prevention Research Center, Isfahan University of Medical Sciences, Isfahan, Iran*

**Keywords:** CD137, Psoriasis, T cell, TNF-alpha, Tumor necrosis factor receptor

## Abstract

**Background & Objective:**

CD137 is a member of the TNF-Receptor family. TNF-alpha antagonists have therapeutic effect in active psoriasis. In this study, the relative frequency of CD137 expression was investigated in the inflammatory cells of psoriasis lesions for the first time.

**Methods:**

The specimens were obtained from pathology department of Al-Zahra hospital from paraffin-embedded skin specimens collected from 2007 till 2016. . A total number of f 64 psoriasis skin specimens and 34 normal skin specimens were reviewed for the diagnosis. Then, the immunohistochemical staining for CD137, CD4, and CD8 was performed.

**Results:**

CD137 expression of dermal inflammatory cells in psoriasis lesions was 11.19±5.5%. Although, in normal skin tissues, CD137 expression was observed in 1.3±3.03% of the inflammatory cells. (*P*=0.001). The relative frequency of the CD137 positive inflammatory cells was significantly higher in the epidermis compared to dermis (epidermis: 31.1%±12.8, dermis 11.1%±5.5). There was no remarkable relation between the CD137 expression rate and the CD4: CD8 ratio.

**Conclusion:**

CD137 as a TNF-alpha receptor has a significant role in pathogenesis of the psoriasis lesions. Therefore, CD137 antagonists can be considered as a novel target for the treatment of incurable psoriasis patients.

## Introduction

Psoriasis is a chronic papulosquamous dermatitis with different ranges of incidence of 0.91 to 8.5% and 0 to 2.1% among adult and children, respectively (1). Although the mean age of onset is around 25 years, people in all age groups can be affected (2). It is typically characterized by erythematous thick silver-white plaques on the scalp, trunk, and extensor surface of extremities. In addition, psoriasis has some histopathological characterizations including epidermal hyperplasia, hyperkeratosis, parakeratosis, spongiform pustules of Kogoj, supra-papillary thinning of granular layer, dermis capillary proliferation, and inflammatory infiltrate in dermis (3). 

Although epidermal hyperplasia and altered keratinocyte differentiation are prominent in this disease, many studies have demonstrated the immune system is the main player in its pathogenesis (4-6). Psoriasis exhibits T cell-mediated autoimmune disease features and provokes through considerable T cells infiltration in the lesions (7). Effective T cells’ activations depend on the interaction of the T cells costimulatory receptors with their ligands on antigen presenting cells. Moreover, co-stimulatory signals are essential for prevention T cells anergy (8). Therefore, one of the most effective treatments against psoriasis is inhibition of T cells and the co-stimulatory receptors. Tumor necrosis factor (TNF) receptor (TNFR) is one of the most well-known classes of co-stimulatory receptors. Many pieces of evidence demonstrated the pivotal role of TNF-alpha in psoriasis pathogenesis like elevated levels of TNF-alpha in both blood and skin lesions at the disease activation time (8, 9). 

CD137 is a member of the TNFR family. Its alternative names are tumor necrosis factor receptor superfamily member 9 (TNFRSF9) and 4-1BB (10). CD137 is an effective co-stimulatory molecule to T lymphocytes. Many studies have demonstrated the undeniable role of CD137 in the regulation of survival and proliferation of T lymphocytes. CD137 is present in immune cells such as activated T and B lymphocytes and monocytes, and several other cell lineages (11). 

CD137 regulates the T cells and behaves as a tumor necrosis factor receptor. Its role can be more prominent in psoriasis due to elevated levels of TNF-alpha in both patients' blood and skin lesions. In this study, for the first time, we decided to investigate CD137 expression in psoriasis skin lesions by immunohistochemical method and evaluate the relationship of the expression with CD4/CD8 ratio. This can lead to a better understanding of the disease pathogenesis and new insights for treatment approaches.

## Materials and Methods

**Normal and Psoriasis Skin Specimens**

This study was a cross-sectional research with backward direction. The purpose of this study was to determine mean of CD137 expression in patients’ skin psoriasis biopsy (40% male, age range, 14-74 years, mean age 40 years) compared with normal skin specimens (46% male, age range, 25-70 years, mean age 43 years). This study was performed at Al-Zahra hospital pathological lab in Isfahan University of Medical Sciences. The specimens were obtained from pathology department of Al-Zahra hospital between 2007-2016 years from paraffin-embedded skin biopsies. The sampling was performed by a simple method and all the patients with skin psoriasis for whom diagnostic biopsy was carried out were included. . All the samples were reviewed by two expert pathologists separately and if there was any discrepancy between them or with clinical data, the sample would be excluded from the study. The psoriasis of the nail was also excluded. For comparison with normal skin, we prepared paraffin embedded blocks from excisional skin sample near to tumoral lesions of the breast and extremities. In addition, these controls were reviewed by two other expert pathologists and if there was any pathological lesions, they were excluded from the study. The histopathological characteristics of the specimens were retrieved from the formal pathology reports and verified by the pathologists. Those included: age, gender, and site of lesions.

**Sample Size**

According to below formula, 64 specimens were calculated as appropriate sample size. We considered statistical confidence interval of 95% (Z_1-α/2_=1.96) and statistical power of 80% (Z_1-β_=0.84). Also, it was assumed that variance in both groups (with psoriasis and normal tissue) for CD137 expression was equal and difference of mean (µ_1_-µ_2_) was 0.7.

$n=\frac{({Z_{1-\alpha /2}+Z_{1-\beta })}^{2}({{\mathrm{SD}}_{1}^{2}+{\mathrm{SD}}_{2}^{2})}^{2}}{{\left({µ}_{1}-{µ}_{2}\right)}^{2}}$

**Processing of Paraffin-embedded Specimens, Immunohistochemical Analysis, and Tissue Microarray**

The samples were sited together in blocks of ten with Tissue Microarray technology (TMAs). The whole sections of skin psoriasis and normal skin tissue samples were sectioned at 0.3 μm thickness. Then, they were deparaffinized in xylene and hydrated in graduated alcohols. Slides were pretreated with 10 mmol/L citrate buffer (pH=6.0). The slides were covered with the anti-CD137 antibody (clone BBK-2; biorbyt, Cambridge) at 1:10 dilution. Tissue peroxidase was inactivated by exposure of the slides by 0.3% H_2_O_2_ in phosphate buffer saline. Slides were developed using the Dako Envision method (Dako, Carpinteria, CA) and were coverslipped with the aqueous-based mounting medium. In addition, immunohistologic staining for CD4 (Clone 4B12, Novocastra Laboratories) and CD8 (C8/144B, Dako) were performed on skin psoriasis blocks separately. Sections from reactive tonsil were utilized as positive control for CD137, CD4, and CD8 staining. The cells with cytoplasmic staining were considered positive.

**CD137, CD4, and CD8 Expression Quantification**

The evaluation of the immunohistochemical staining was performed by light microscopy using a 40x objective lens to determine the stained cells at 10-HFP. The stained sections were reviewed by two independent observers and a consensus regarding controversial cases was reached with the aid of a double-headed microscope. For CD137, all stained cells in dermal and epidermal (in neutrophils) were counted separately. All inflammatory cells in dermis and PMNs in the epidermis were also counted. CD137 positive cells were divided upon inflammatory cells in dermis and neutrophils in the epidermis to achieve the relative frequency of CD137 expression in dermis and epidermis, respectively. CD4 positive and CD8 positive cells also were counted in the dermis and divided to obtain of CD4:CD8 ratio. 

**Statistical Analysis**

SPSS software version 16 (SPSS Inc., Chicago, Ill., USA) was used to determine any relationship between data. To compare the mean of relative frequency of CD137 expression in both groups, the T-student analysis was utilized and to determine the relationship between CD137 expressions with CD4:CD8 ratio, correlation analysis, and correlation coefficient value were used. The results were considered to be statistically significant if the P-value for the null hypothesis was <0.05. 

## Results

**Assessment of the CD137 Expression in Inflammatory Cells of Normal Skin in Comparison with the Psoriasis Lesions**

**Fig. 1:**
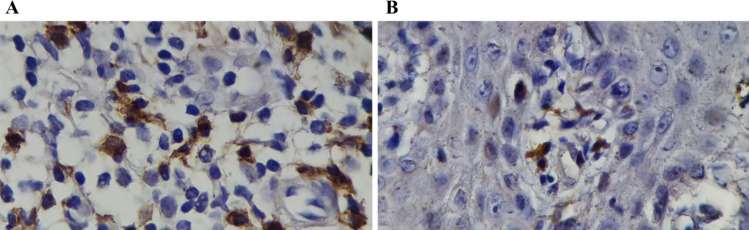
CD137 cytoplasmic immunoreactivity in inflammatory cells of a psoriatic lesion in A) within the dermis and B) at the dermo-epidermal junction

**Fig. 2:**
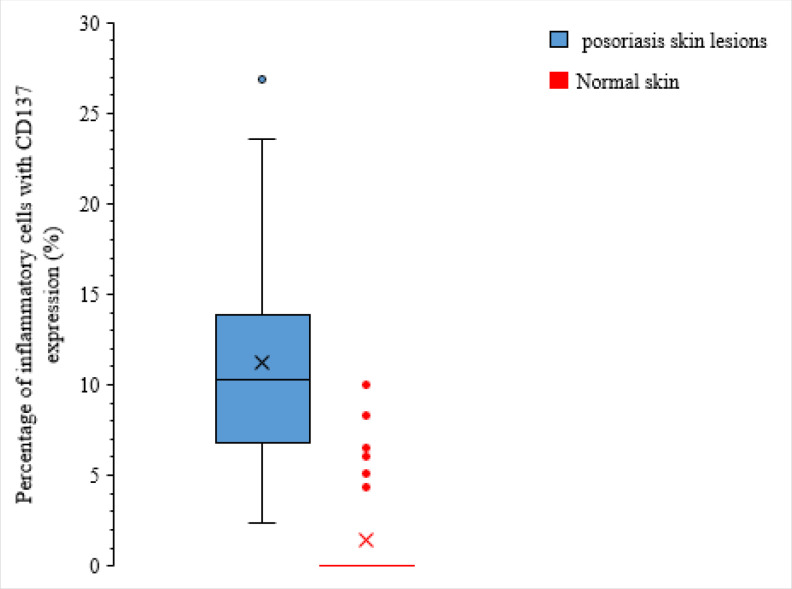
The relative frequency of the CD137-positive inflammatory cells in the psoriasis skin lesions and normal skin specimens** (P=0.001)**

A total number of 64 specimens from patients’ skin lesions with confirmed psoriasis diagnosis were stained for CD137 by IHC. Figure 1 shows CD137 cytoplasmic expression in dermis and dermo-epidermal of the inflammatory cells. As Figure 2 illustrates, a significant number of CD137 expressing inflammatory cells were observed at the psoriasis lesions. However, CD137 expression was extremely rare in the normal skin specimens. In the mean, 11.1±5.5% of inflammatory cells within the psoriasis skin lesions exhibited positive cytoplasmic CD137 expression, but this was 1.3±3.03% for normal skin specimens (*P*=0.001). Therefore, considerable CD137 positive inflammatory cells are attracted to the psoriasis skin lesions which can play a prominent role in the pathogenesis (Table 1).

**Table 1:** The relative frequency of the CD137-positive inflammatory cells in the psoriasis skin lesions and normal skin specimens

	Mean percent of CD137 expression ± SD
Normal skin	1.3 ± 3.03%
Psoriasis lesion	11.19 ± 5.5%
	*P*=0.001

**Assessment of the CD137 Expression in Inflammatory Cells of the Dermis and Epidermis in the Psoriasis Lesions**

To evaluate the main site of the CD137 positive action, the relative frequency of the inflammatory cells’ CD137 expression were investigated within the dermis and epidermis of psoriasis skin lesions. A significant CD137 expression in the epidermis compared with dermis (*P*=0.024) was observed. However, inflammatory cells in epidermis were only seen in 23 psoriasis lesions for this comparison (Table 2).

.

**Table 2:** The relative frequency of the CD137 expressing inflammatory cells in the dermis and epidermis of the psoriasis lesions

	The mean relative frequency of CD137-positive inflammatory cells (%)	The median relative frequency of CD137-positive inflammatory cells (%)
The epidermis of psoriasis skin lesions (23 cases)	31.1 % ± 12.8	34.6 %
The dermis of psoriasis skin lesions (64 cases)	11.1 % ± 5.5	10.3 %
*P*=0.024		

**The Relationship Between CD137 Expression and CD4/CD8 Ratio**

The CD4/CD8 ratio is an important marker to differentiate acute and chronic lesions of psoriasis. The median relative frequency of the CD137 positive inflammatory cells was selected to divide the specimens into two groups including specimens with lower or higher expression of CD137 than the median expression rate. This classification was done for both dermis and epidermis data (Table 3). When we compared each row with t-student analysis, we did not observe any significant differentiation between CD4:CD8 expression in 2 group, both in dermis and epidermis (*P*=0.621 and *P*=0.120 respectively) In addition, Chi-squared analysis did not show a significant relationship between CD137 expression rate and CD4 to CD8 ratio both in dermis and epidermis (*P*=0.984 and *P*=0.821 respectively).

**Table 3:** Evaluation of the CD137-positive cells relative frequency relation with the CD4/CD8 ratio

	The relative frequency of CD137-positive inflammatory cells (%) in the dermis			The relative frequency of CD137-positive inflammatory cells (%) in the epidermis		
	**Classification**			**Classification**		
	< 10.3 %	≥10.3 %		< 34.6 %	≥34.6 %	
Mean CD137 expression	6.8 % ± 2.6	15.9 % ± 4.4	*P*<0.001 t-student	19.7 % ± 8.7	42.4 % ± 3.3	*P*<0.001 t-student
CD4/CD8 ratio	0.14 % ± 0.2	0.09 % ± 0.19	*P*=0.621 t-student	0.04 % ± 0.03	0.17 % ± 0.4	*P*=0.120 t-student
	*P*=0.984 (Chi-square)			*P*=0.821 (Chi-square)		

## Discussion

Psoriasis is a common chronic skin disorder which is classically characterized by silver scaly erythematous papules and plaques (1, 12). Although early notions for the psoriasis pathogenesis were attracted on hyperproliferation of the keratinocytes, now the immune system dysregulation is more susceptible to the disease pathogenesis (12). Totally, many cells (like T cells and neutrophils) and cytokines such as TNF-alpha are involved in psoriasis development as a known autoimmune disease (13, 14). 

TNF-alpha has gained much attention in psoriasis pathogenesis due to its elevated levels in the blood and skin lesions of the patients (15). The CD137 is a member of TNF receptor family and a potent co-stimulator of CD8+ T cells. The interaction of this T cells receptor with their specific activators on the antigen-presenting cells is necessary for the T cells activation. Many studies have demonstrated the significant effect of agonistic CD137-specific monoclonal antibody for provoking an anti-tumor response by the reinforcement of weak CD8+ T cells (8, 16). On the other hand, CD137 monoclonal antibodies with agonistic effect over this receptor exhibit inhibition of the T cell-dependent humoral response. This can be attributed to CD4+ T cell function distribution (8). Also, CD137 is constitutively expressed by non-active neutrophils; these cells penetrate into the epidermis in psoriasis (17). Therefore, CD137 has an undeniable role in the orchestrating the immune reactions and this fact can be extended to the psoriasis skin lesions, too. According to our results, the high relative frequency of the CD137-positive inflammatory cells is present at the psoriasis skin lesions which are absent in normal skin. These cells like the above-mentioned facts can have a vital function in these lesions and their targeting for therapeutic goals can disrupt the pathologic cycle in the psoriasis skin lesions. 

The expression of CD4-positive lymphocytes, as well as CD8-positive lymphocytes and macrophages, were found to be significantly increased in the psoriasis patients' skin epidermis and dermis in comparison with healthy skin (18). In acute and chronic phases of the disease, the CD4-positive and CD8-positive cells were dominant among the infiltrating cells, respectively. This fact causes a remarkable decrease in CD4/CD8 ratio in skin lesions in the chronic phase in both the dermal papillae and the epidermis (19). Therefore, the CD4:CD8 ratio is a diagnostic criterion to determine the acute and chronic phase of the disease. However, it was found that there was no meaningful relationship between the CD137 expression rate and the CD4/ CD8, ratio in this study. This fact shows that CD137 has a role in acute and chronic phase of the disease and can be considered for therapeutic roles in entire phase of the disease. 

There is a variety of methods for treatment of the moderate-to-severe chronic psoriasis plaque including well-known anti-TNF agents (e.g. etanercept, adalimumab, and infliximab) (20). Although these immunotherapeutic agents are highly effective in the treatment of skin and joints psoriasis, they neutralize the effects of TNF on different cell types while it can be vital for the beneficial immune responses (21). TNF-alpha receptors improved our understanding of disease mechanisms and resulted in the development of new small molecules and antibodies that help to control the chronic inflammation with low side effects. However, with increasing, broader, and prolonged use of anti TNF-alpha agents, some patients can show adverse reactions including a wide spectrum of dermatological conditions. The majority of these lesions are identified as psoriasiform (paradoxical psoriasis induced TNF-alpha agent) with different morphologies consisting of pustular psoriasis, guttate; and erythrodermic or inverted psoriasis (22, 23). Given the presence of different types of TNF-alpha receptors among inflammatory cells, these findings point us to the use of more specific drugs to reduce the adverse effects of current drugs for treatment of psoriasis. This study is the first study that demonstrates enhanced relative frequency of CD137-positive inflammatory cells in psoriasis skin lesion compared with the normal skin. This study not only enhances our knowledge about the pathogenesis of the psoriasis skin lesions but also can be the first report to introduce CD137 as a novel target for treatment. 

##  Conclusion

Psoriasis is a chronic inflammatory dermatological and disabling disease. The immune system plays a pivotal role in its pathogenesis. Many pieces of evidence demonstrated the crucial role of TNF-alpha in psoriasis pathogenesis, like elevated levels of TNF-alpha in both blood and skin lesions at the disease activation time. In this study for the first time, we demonstrated the considerable role of CD137 expressing cells in the pathogenesis of the psoriasis skin lesions. The expression of CD137 is the same in acute and chronic phase of the disease. Therefore, CD137 antagonists can be considered as a novel target for the treatment of incurable psoriasis patients in entirely period of the disease. 

##  Financial Support

This work was financially supported (Grant No: 395468 and No: 195113) by the Isfahan University of Medical Sciences, Isfahan, Iran.

## Conflict of Interest

The authors declare no conflict of interest.
